# Solitary Renal Metastases From Stage IA Primary Lung Adenocarcinoma With Co‐Alteration of *EGFR*, *RB1*, and *MAP3K1*: A Case Report

**DOI:** 10.1111/crj.70018

**Published:** 2024-10-09

**Authors:** Zhu Qin, Chen Xin, He Zhenzhen, Xie Liang, Yi Wei, Li Shuben

**Affiliations:** ^1^ Department of Radiation Oncology The First Affiliated Hospital of Guangzhou Medical University Guangzhou China; ^2^ Department of Pathology The First Affiliated Hospital of Guangzhou Medical University Guangzhou China; ^3^ Department of Radiation Oncology Guangxi Academy of Medical Sciences & the People's Hospital of Guangxi Zhuang Autonomous Region Nanning China; ^4^ Department of Thoracic Surgery The First Affiliated Hospital of Guangzhou Medical University Guangzhou China

**Keywords:** *EGFR*, *RB1*, and *MAP3K1* co‐alteration, lung adenocarcinoma, renal metastasis

## Abstract

We report a case of 59‐year‐old female with solitary bilateral renal metastases after surgery of stage IA primary lung adenocarcinoma who underwent next‐generation sequencing (NGS) of both lesions. The patient received right upper lobectomy and lymph node dissection, which revealed primary invasive lung adenocarcinoma (pT1cN0M0, stage IA3). Two years following this, positron emission tomography–computed tomography (PET/CT) revealed multiple masses in both kidneys without other distant metastases, and ultrasonography‐guided puncture biopsy indicated the presence of metastatic lung adenocarcinoma. The NGS of both the primary and metastatic lesions revealed the co‐alteration of epidermal growth factor receptor (*EGFR*), RB transcriptional corepressor 1 (*RB1*), and mitogen‐activated protein kinase kinase 1 (*MAP3K1*), which is potentially associated with the risk of renal metastasis in early postoperative non‐small cell lung cancer.

## Graphical abstract

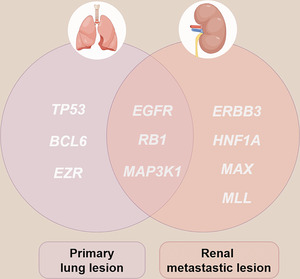

## 1 Introduction

About 47.3% of patients with non‐small cell lung cancer (NSCLC) may experience metastasis during the course of the disease, which more commonly occurs in the bones (34.3%), lungs (32.1%), brain (28.4%), adrenal glands (16.7%), and liver (13.4%) [1]. In contrast, metastasis to the kidneys is rare, and instances of pathology‐confirmed solitary renal metastasis are exceptionally rarer, typically documented in case reports [2, 3, 4]. A study involving 933 postoperative stage I NSCLC patients showed that only 3.6% of those with stage IA had metastasis to a single organ, and no renal metastases were observed [5]. To our knowledge, fewer than 10 documented cases exist in the literature confirming renal metastasis in patients with postoperative stage IA NSCLC, resulting in limited explicit prognostic data [2, 6]. However, it is evident that patients in postoperative stage IB‐III experience poor prognoses, which also underscores the critical need for identifying molecular markers associated with early renal metastasis in NSCLC [7, 8, 9]. We searched the patients with confirmed renal metastases through pathology records from January 1, 2019, to July 1, 2023, in the pathology database of our institute (The First Affiliated Hospital of Guangzhou Medical University, Guangzhou, China); the clinicopathologic features of the 10 retrieved cases are listed in Table 1. Notably, these individuals mainly included NSCLC, with an average age of 57.6 (95% CI: 50.1–65.1) years. Remarkably, the earliest TNM stage observed was T1N0M0 IA. Metastasis may occur 44 (95% CI: 0.7–82.3) months after the initiation of standard treatment, and we found no unilateral or bilateral differences. In this case report, we focus on the patient with solitary renal metastases from stage IA lung adenocarcinoma after radical resection of the right upper lobe who underwent NGS for both lesions. We also attempted to verify our hypothesis using the bioinformatics database.

**TABLE 1:** The clinicopathologic feature of 10 patients with renal metastasis.

	Age (years)	Gender	TNM stage	Primary lesion	Pathology	Treatment	Renal metastasis		
							Metastatic time (months)	Location	Treatment
Patient 1^a^	59	Female	T1cN0M0 IA3	Lung	Adenocarcinoma	Operation	23	Bilateral	Targeted therapy
Patient 2	57	Male	T2N0M0 IB	Lung	Large cell carcinoma	Operation + adjuvant chemotherapy	23	Unilateral	Operation + adjuvant chemotherapy
Patient 3	47	Male	T2N0M0 IB	Lung	Adenocarcinoma	Operation + adjuvant targeted therapy + chemoradiotherapy	192	Unilateral	—
Patient 4	72	Male	T3N0M0 IIB	Lung	Squamous cell carcinoma	Operation + adjuvant chemotherapy	18	—	—
Patient 5	38	Male	T1N3M0 IIIB	Lung	Adenocarcinoma	Operation + adjuvant targeted therapy	27	Unilateral	Operation + adjuvant chemotherapy and targeted therapy
Patient 6	50	Male	T4N3M0 IIIC	Lung	Large cell carcinoma	Chemotherapy + radiotherapy	26	Bilateral	Operation + adjuvant chemotherapy and immunotherapy
Patient 7	70	Male	T3N3M0 IIIC	Lung	Adenosquamous carcinoma	Operation + adjuvant chemotherapy	48	Bilateral	Operation + adjuvant chemotherapy and immunotherapy
Patient 8	57	Male	T4N3M0 IIIC	Lung	Squamous cell carcinoma	Neoadjuvant chemotherapy + operation + radiotherapy	26	Unilateral	Operation + adjuvant targeted therapy
Patient 9	52	Male	T3N2M0 IIIB	Esophagus	Squamous cell carcinoma	Operation + adjuvant chemotherapy	13	Bilateral	—
Patient 10	68	Male	TxN0M1a IVA	Cancer of unknown primary	Adenocarcinoma	—	—	—	—

*Note:* —, lost follow‐up or just diagnosed; +, sequential therapy; “and”, concurrent therapy.

^a^ The case reported in this paper.

## 2 Case Report

**FIGURE 1:**
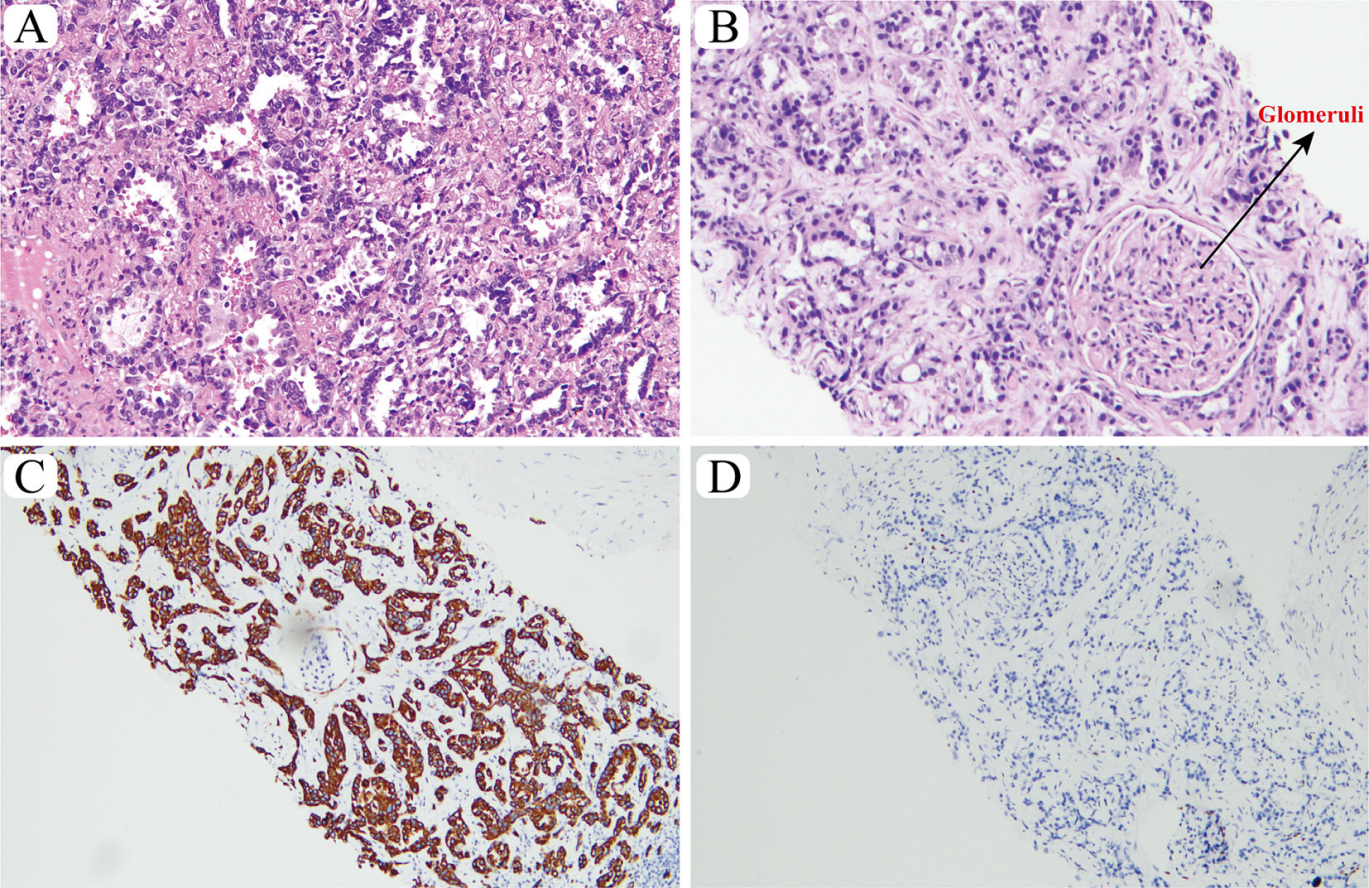
The pathology, microscopic appearance, hematoxylin–eosin (HE) staining. (A) Primary lung lesion (magnification 200×). (B) The renal biopsy tissue (magnification 200×). (C) The renal biopsy tissue was positive for CK7. (D) The renal biopsy tissue was negative for PAX‐8.

A 59‐year‐old female was found to have an uneven nodular shadow in the right upper lung lobe on a chest enhanced computed tomography (CT) scan during a health examination on May 18, 2021. The mass was located in the anterior segment of the upper lobe of the right lung, with an approximate size of 2.3 × 1.6 × 1.7 cm, with no obvious enlargement of the hilar or mediastinal lymph nodes. Additionally, contrast‐enhanced magnetic resonance imaging (MRI) of the head and CT of the abdomen did not reveal any evidence of distant metastases. On May 19, 2021, the patient underwent video‐assisted thoracic surgery (VATS) for radical resection of the right upper lobe and lymph node dissection in the Thoracic Surgery Department of the First Affiliated Hospital of Guangzhou Medical University in China. Postoperative pathology confirmed invasive lung adenocarcinoma and negative margins (Figure 1A), with no metastatic lymph nodes detected (0/5; pT1cN0M0, stage IA3). The immunohistochemical staining was positive for TTF‐1, CK7, C‐Met, PI3K, and negative for ALK (D5F3), ROS1 (D4D6). After surgery, the patient underwent regular follow‐up but did not receive any postoperative adjuvant therapy.

**FIGURE 2:**
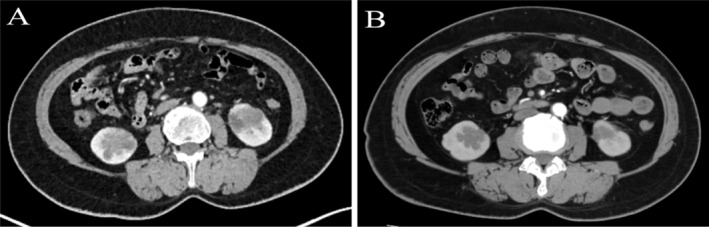
The enhanced CT scan of renal metastases. (A) Before targeted therapy; (B) 6 weeks after targeted therapy. Tumor response was judged as partial response.

However, on April 8, 2023, an [^18^F] fluorodeoxyglucose (18F‐FDG) PET/CT scan revealed multiple renal masses (the largest one was located in the lower pole of the right kidney, measuring approximately 3.5 × 2.6 × 4 cm in size) (Figure 2A), with no urological symptoms such as waist pain and hematuria. Peritoneal metastatic lymph nodes were detected, with no evidence of other distant metastases. Subsequently, a puncture biopsy of the largest right renal tumor was conducted under US, and the immunohistochemistry staining was positive for CK7, Napsin A and TTF‐1, while the renal‐origin marker PAX‐8 was negative; taken together, these findings suggested metastatic lung adenocarcinoma (Figure 1B).

Following this, we conducted NGS on specimens from both the primary lung lesion and the largest renal metastasis. The findings revealed a mutation frequency of 47.1% for *EGFR* p.L858R (exon 21), 32.5% for *RB1* p. N849Ifs24 (exon 25), and 26.4% for *MAP3K1* p.Q1247 (exon 15). Meanwhile, in the renal metastasis, we observed a mutation frequency of 26.0% for *EGFR* p.L858R (exon 21), 15.4% for *RB1* p. N849Ifs24 (exon 25), and 18.6% for *MAP3K1* p.Q1247 (exon15) (Table 2). Furthermore, for both lesions, programmed cell death‐ligand 1 (PD‐L1) expression was negative, tumor mutational burden (TMB) was low, and there was microsatellite stability. According to the NGS results, the patient received targeted therapy with osimertinib (80 mg once a day) and was subsequently followed up with enhanced CT scan after 6 weeks (Figure 2B), which revealed a partial response. Following this, the patient continues to receive targeted treatment with osimertinib and remains alive to date.

**TABLE 2:** The next‐generation sequencing of primary lung adenocarcinoma and renal metastasis.

	Primary lung adenocarcinoma		Renal metastasis	
	Gene	Functional areas change and mutation frequency/copy coefficient	Gene	Functional areas change and mutation frequency/copy coefficient
Somatic mutations	***EGFR*** ***RB1*** ***MAP3K1*** *TP53* *BCL6* *EZR*	EX21 p.L858R (47.1%) EX25 p.N849Ifs24 (32.5%) EX15 p.Q1247 (26.4%) deletion (0.6) EX3 p.R13H (1.1%) EX13 p.D581N (1.0%)	***EGFR*** ***RB1*** ***MAP3K1*** *ERBB3* *HNF1A* *MAX* *MLL*	EX21 p.L858R (26.0%) EX25 p. N849Ifs24 (15.4%) EX15 p.Q1247 (18.6%) EX28 p.E1193K (11.5%) EX7 p.Q444H (8.2%) EX4 p.R75 (7.7%) EX27 p.S2956 (2.9%)
Germline mutations	—	—	—	—

*Note:* EX, exon; — no mutations.

## 3 Discussion

Renal metastasis of primary lung cancer is rare due to the renal relatively strong resistance to malignant tumor metastasis. Bracken and colleagues conducted an autopsy analysis that included 11328 cancer‐related deaths from 1944 to 1974, revealing a 1.98% incidence of renal metastasis from primary lung cancer [10]. Moreover, most of the literature concerning renal metastasis consists of case reports, with the majority of cases being stages II–IV [8, 11, 12, 13]. To our knowledge, fewer than 10 documented cases exist in the literature confirming renal metastasis in patients with postoperative stage IA NSCLC [2, 6]. According to the clinicopathologic feature analysis from our institute (Table 1), only one patient with postoperative renal metastasis of lung cancer was stage IA. We hypothesize that there might be some specific genetic factors driving early metastasis to the kidneys in this patient.

Previous studies have indicated that different genetic characteristics may lead to different biological behaviors of tumor, including early metastasis. Deng et al. discovered a notable increase in the occurrence of brain and bone metastases in patients with completely resected *EGFR* mutational lung adenocarcinoma [14]. Moreover, a large‐scale genomic map illustrating organotropic trends in lung adenocarcinoma metastasis suggested a strong correlation between *TP53* alterations and a higher metastatic burden, as well as a greater frequency of lymph node metastases. Interestingly, among those with co‐alteration of *TP53*, *EGFR* was observed only in the metastatic group [15]. It has also been discovered that *RB1* can interact with E2F transcription factor 1, thereby influencing the process of epithelial–mesenchymal transition and metastasis in NSCLC [16]. Through testing with comprehensive genomic profiling of 3035 patients with NSCLC, Huang et al. also observed a significant enrichment of *TP53* and *RB1* in individuals with brain metastases [17].

Furthermore, many studies have suggested that the co‐alteration of *EGFR*, *TP53*, and *RB1* within the tumor may relate to the distant metastases of brain. The co‐alteration of the tumor suppressors *TP53* and *RB1* has been commonly observed in small‐cell lung cancer (SCLC), which often exhibits early metastasis [18]. In one study, it was reported that approximately 5% of patients with NSCLC had co‐alteration in *EGFR*, *TP53*, and *RB1* and that these patients exhibited a unique risk of histologic transformation into SCLC [19]. Notably, all of these patients with co‐alteration of *EGFR*, *TP53*, and *RB1* had metastatic disease, especially metastasis of the brain. Approximately 65% of these patients developed brain metastasis during the course of their disease, with 42% already exhibiting brain metastasis at the time of diagnosis, and an additional 23% developing the metastases during treatment [19]. Meanwhile, Marcoux and colleagues also reported a high rate of central nervous system (CNS) involvement in patients with co‐alteration in *EGFR*, *TP53*, and *RB1*. In their study, 94% of patients with available follow‐up radiographic data after SCLC transformation experienced progression in the CNS [20].

Similarly, in this case with solitary renal metastases, co‐alteration of *EGFR*, *RB1*, and *MAP3K1* was observed in both the primary and metastatic lesions. As is widely recognized, *MAP3K* is a key factor of *MAPK* signaling pathway, in which *MAP3K* can activate *MAP2K* and ultimately activate *MAPK*. Previous studies [21, 22, 23] have demonstrated that *MAP3K1* can significantly promote cell growth, migration, and invasion of breast cancer by upregulating matrix metalloproteinase‐9 (MMP‐9) and urokinase plasminogen activator (u‐PA), which can degrade the extracellular matrix and basement membrane of blood vessels. Considering this evidence cumulatively, we hypothesize that co‐alteration of *EGFR*, *RB1*, and *MAP3K1* may be the specific genetic driver to promoting the process of renal metastasis in early NSCLC. In order to further validate this hypothesis, we downloaded the gene expression quantification data and clinical information of 480 patients with lung adenocarcinoma or lung squamous cell carcinoma in The Cancer Genome Atlas (TCGA) database (https://portal.gdc.cancer.gov/) using TCGA biolinks, but no patient with co‐alteration of *EGFR*, *RB1*, or *MAP3K1* was found; however, this may be attributable to the rarity of renal metastasis.

The National Comprehensive Cancer Network (NCCN) Guidelines, Version 4.2024, for NSCLC recommend osimertinib as an adjuvant treatment option for patients with completely resected (R0) stage IB to IIIA NSCLC who have undergone adjuvant chemotherapy or are unsuitable for it [24]. For patients with postoperative stage IA, the guidelines recommend postoperative observation and regular follow‐up. However, up to 16% of patients with postoperative stage IA NSCLC still experience recurrence within a median 4‐year follow‐up period [25]. A recent study has demonstrated that adjuvant EGFR‐TKIs for patients with stage IA can increase the 5‐year disease‐free survival rate from 84.5% to 100% [26]. Additionally, to our knowledge, this is the first documented case in the literature of a patient with NSCLC with co‐alteration of *EGFR*, *RB1*, and *MAP3K1*, who developed solitary renal metastases after being completely resected and postoperative observation. This may suggest that for postoperative patients with high‐risk factors in stage IA, such as the co‐alteration of *EGFR*, *RB1*, and *MAP3K1*, adjuvant targeted therapy may be needed.

In this case report, we present evidence supporting a potential relationship between co‐alteration of *EGFR*, *RB1*, and *MAP3K1* and early renal metastasis and hope greater attention can be brought to bear on this type of co‐alteration. Concurrently, form the 10 patients with confirmed renal metastases through pathology in our institute, we observed the epidemiological characteristics of these patients, predominantly middle‐aged men with NSCLC. Additionally, no significant differences were identified in TNM stage, pathology type, or the presence of unilateral or bilateral metastases among these patients. It is worth noting that postoperative adjuvant therapy might contribute to delaying the occurrence of renal metastasis. However, due to the limited number of patients, comprehensive and dependable characteristics need to be explored by further large‐scale clinical trials.

## Author Contributions

All authors read and approved the final manuscript. Z.D.: study conception, clinical data curation and writing – original draft preparation. C.X.: bioinformatics database validation and draft writing participation. H.Z.Z.: pathological interpretation and list collection. X.L.: clinical data curation and draft writing participation. Y.W. and L.S.B.: study conception and writing – review and editing. All authors are in agreement with the content of the manuscript.

## Ethics Statement

The study was approved by the Ethics Committee of The First Affiliated Hospital of Guangzhou Medical University ([2023]MR‐44‐23‐025996). All procedures performed in this study were in accordance with the ethical standards of the institutional and/or national research committees and with the Helsinki Declaration (as revised in 2013).

## Conflicts of Interest

The authors declare no conflict of interest.

## Data Availability

Data sharing not applicable to this article as no datasets were generated or analysed during the current study.
